# Contribution of *Lactobacillus iners* to Vaginal Health and Diseases: A Systematic Review

**DOI:** 10.3389/fcimb.2021.792787

**Published:** 2021-11-22

**Authors:** Nengneng Zheng, Renyong Guo, Jinxi Wang, Wei Zhou, Zongxin Ling

**Affiliations:** ^1^ Department of Gynecology and Obstetrics, The First Affiliated Hospital, College of Medicine, Zhejiang University, Hangzhou, China; ^2^ Department of Laboratory Medicine, The First Affiliated Hospital, College of Medicine, Zhejiang University, Key Laboratory of Clinical In Vitro Diagnostic Techniques of Zhejiang Province, Hangzhou, China; ^3^ Collaborative Innovation Center for Diagnosis and Treatment of Infectious Diseases, State Key Laboratory for Diagnosis and Treatment of Infectious Diseases, National Clinical Research Center for Infectious Diseases, The First Affiliated Hospital, School of Medicine, Zhejiang University, Hangzhou, China; ^4^ Institute of Microbe & Host Health, Linyi University, Linyi, China

**Keywords:** bacterial vaginosis, dysbiosis, *Lactobacillus iners*, sexually transmitted infections, vaginal microbiota

## Abstract

*Lactobacillus iners*, first described in 1999, is a prevalent bacterial species of the vaginal microbiome. As *L. iners* does not easily grow on de Man-Rogosa-Sharpe agar, but can grow anaerobically on blood agar, it has been initially overlooked by traditional culture methods. It was not until the wide application of molecular biology techniques that the function of *L. iners* in the vaginal microbiome was carefully explored. *L. iners* has the smallest genome among known *Lactobacilli* and it has many probiotic characteristics, but is partly different from other major vaginal *Lactobacillus* species, such as *L. crispatus*, in contributing to the maintenance of a healthy vaginal microbiome. It is not only commonly present in the healthy vagina but quite often recovered in high numbers in bacterial vaginosis (BV). Increasing evidence suggests that *L. iners* is a transitional species that colonizes after the vaginal environment is disturbed and offers overall less protection against vaginal dysbiosis and, subsequently, leads to BV, sexually transmitted infections, and adverse pregnancy outcomes. Accordingly, under certain conditions, *L. iners* is a genuine vaginal symbiont, but it also seems to be an opportunistic pathogen. Further studies are necessary to identify the exact role of this intriguing species in vaginal health and diseases.

## Introduction

The vaginal microbiome plays an important role in determining human vaginal health. Using high-throughput metagenomic and 16S rRNA sequencing, over 250 bacterial species have been identified in the human vagina (Fredricks et al., 2005; Chen et al., 2020). Among them, *Lactobacillus* is the most frequently detected microorganism in the healthy vagina, and this includes *Lactobacillus crispatus*, *Lactobacillus iners*, *Lactobacillus jensenii*, and *Lactobacillus gasseri* (Alonzo Martinez et al., 2021). For decades, *Lactobacillus* species have been regarded as beneficial to the vaginal econiche by preventing the invasion of pathogens through the production of organic acids, hydrogen peroxide (H_2_O_2_), bacteriocin, and other antimicrobial compounds (Petrova et al., 2015). Predisposing factors, such as menstruation, pregnancy, sexual practices, vaginal douching, and uncontrolled use of antibiotics, can rapidly alter the microbial community (Chee et al., 2020). A disruption of the vaginal ecosystem is characterized by the depletion of *Lactobacillus* species and the overgrowth of non-*Lactobacillus* microbes. Typically, the overgrowth of anaerobic bacteria can result in aberrant conditions, such as bacterial vaginosis (BV) and sexually transmitted infections (STIs), as well as pregnancy-related complications (Fredricks et al., 2005; Bautista et al., 2016; Chang et al., 2020).

Since the advent of metagenome sequencing techniques, *L. iners* has been recognized as the most prevalent *Lactobacillus* species in the vaginal econiche (Spear et al., 2011; Campisciano et al., 2020). This species has been initially overlooked in past bacteriologically-based studies because of its fastidious requirements and inability to grow on de Man-Rogosa-Sharpe agar (MRS), a selective culture medium that isolates vaginal *Lactobacill*i (Falsen et al., 1999). Furthermore, *L. iners* has very unique characteristics compared with other symbiotic *Lactobacillus* species in the vaginal econiche (Vaneechoutte, 2017). Most vaginal *Lactobacillus* species exert a protective effect and play a role in the resistance of the vaginal tract to colonization by pathogens. However, the relationship between *L. iners* and vaginal health is somewhat complicated and ambiguous (Petrova et al., 2017). This review aims to present the overall characteristics, an overview of different arguments, and the dual roles of *L. iners* in the vaginal econiche.

## Characteristics of *L. iners*

### Culture Characteristics and Gram-Staining Properties

*L. iners* was first described by Falsen et al. in 1999 in vaginal and urinary tract specimens (Falsen et al., 1999). This species had escaped our attention for a long time due to its inability to grow on MRS agar under the same culture conditions as other *Lactobacillus* species (De Backer et al., 2007). Nevertheless, *L. iners* is characterized by small, smooth, circular, translucent, and non-pigmented colonies after 24 h of anaerobic incubation on blood agar (Falsen et al., 1999). Indeed, most *L. iners* isolates can grow on MRS agar upon the addition of 1–5% sheep and human blood (Vaneechoutte, 2017). In addition, Yoshimura et al. demonstrated that *L. iners* can grow on MRS agar under anaerobic conditions for a period of at least 7 days, which is evidently longer than that of other *Lactobacillus* species. In MRS broth with 0.5% cysteine as the reducing agent, which created the anaerobic conditions, *L. iners* grows slowly to its highest concentration of only 10^7^ CFU/ml and then growth decreases after 12 h (Yoshimura et al., 2020).

*L. iners* was initially believed to be a Gram-positive, rod-shaped, non-spore-forming, and facultative anaerobic bacterium (Falsen et al., 1999). However, several studies have reported that, unlike other *Lactobacillus* species, *L. iners* does not always clearly stain as Gram-positive, and it seems to have a coccobacillary rather than a bacillary morphology (De Backer et al., 2007; Lebeer et al., 2008). Yoshimura et al. reported that *L. iners* was mostly Gram-negative with a very short rod shape and weak acid resistance, as it was non-viable in pH 3 medium (Yoshimura et al., 2020). This may be the reason why *L. iners* was initially overlooked by culture and microscopy methods. By transmission electron microscopy, Kim et al. revealed that the peptidoglycan (PG) layer in the cell wall of *L. iners* was thin enough to give an apparent Gram-negative morphology (Kim et al., 2020). This morphological characteristic and Gram-staining property of *L. iners* are clinically very important to consider, as Nugent scoring, which is based on the Gram-staining of vaginal smears, remains a common diagnostic tool in the assessment of vaginal health (Wang et al., 2021). The Gram-negative property of *L. iners* masks the fact that it is a *Lactobacillus* species and this may lead to the misdiagnosis of BV, which is a condition characterized by the depletion of *Lactobacillus* species in Gram-stained vaginal smears under microscopy (Vaneechoutte, 2017). This may help explain why as many as 50% of women diagnosed with BV by the Nugent score are asymptomatic (Klebanoff et al., 2004).

### Genome Size and Function

*L. iners* has the smallest genome of ∼1.3 Mbp on a single chromosome among the *Lactobacillus* species identified so far, with its pangenome count of 2300 genes and average GC content of ~33.3% (France et al., 2016). This low genome size is comparable to those of human symbionts and parasites, and is strongly indicative of a more parasitic, host-dependent lifestyle (Petrova et al., 2017). The genome of *L. iners* seems to have undergone rapid evolution events that resulted in large-scale gene loss and genome reduction, as well as the acquisition of genes, such as iron-sulfur genes, for specific adaption to the vaginal econiche (Macklaim et al., 2011).

Comparative genome analysis revealed that *L. iners* had a severely reduced number of genes related to carbohydrate and amino acid metabolism, whereas it maintained conserved genes for largely core metabolic proteins and membrane transport genes for essential compounds from the host or the community (Macklaim et al., 2011; Kim et al., 2020). Three potential core genes (inerolysin, ZnuA, and hsdR) were identified to be closely related to the specific adaption of *L. iners* to the vaginal environment (Kwak et al., 2020). Among them, inerolysin is an unusual pore-forming cholesterol-dependent cytolysin that is active in the acidic vaginal environment and creates aqueous pores within the cell membrane. It may be one of the essential *L. iners* genes required to stably obtain nutrients from the host (Rampersaud et al., 2011; France et al., 2016). High-affinity zinc uptake requires the binding protein ZnuA type I (ZnuA), which is essential for metal ion homeostasis in *L. iners*. ZnuA may be a key mediator of strong adhesion of *L. iners* to vaginal epithelial cells (Gabbianelli et al., 2011; McMillan et al., 2013). Type I restriction enzyme R protein (hsdR) was suggested to be involved in the defense mechanism against bacteriophage infection during BV (Miller-Ensminger et al., 2018). In addition, *L. iners* contains genes that encode all enzymes directly involved in PG synthesis and hydrolysis (Kim et al., 2020). The unique and thin PG layer of *L. iners* cell membranes may absorb nutrients or secrete proteins more easily than other *Lactobacillus* species, which can provide essential nutrients or respond to rapid changes in the vaginal environment (Kim et al., 2020).

### Ability to Produce Lactic Acid and H_2_O_2_

*Lactobacillus* species are the main lactic acid-producing bacteria in the vagina, and they reduce the vaginal pH and restrict the growth of potentially harmful bacteria (Jang et al., 2019). Nevertheless, this lactic acid-producing ability is different from the main *Lactobacillus* species found in the vaginal microbiome (Godovalov et al., 2019). *L. crispatus*, *L. gasseri*, and *L. jensenii* can produce D- and L-lactic acid by fermenting glycogen, whereas *L. iners* can produce only L-lactic acid because it lacks the gene that codes for D-lactate dehydrogenase in its genome (France et al., 2016). Because of the almost complete absence of D-lactic acid, the L/D lactic acid ratio is highest in *L. iners*. Witkin et al. reported that the isomers of lactic acid have different effects on the host immune system (Witkin et al., 2013). The L/D lactic acid ratio in the vagina may elevate extracellular matrix metalloproteinase inducer (EMMPRIN) and subsequently activate matrix metalloproteinase-8 (MMP-8), which facilitates the breakdown of the extracellular matrix, helps bacteria transverse the cervix, and initiates upper genital tract infections (Beghini et al., 2015). Additionally, D-lactic acid has been reported to have a greater inhibitory effect on exogenous bacteria than L-lactic acid (Tachedjian et al., 2017). Therefore, it seems that L-lactic acid renders *L. iners* less effective in preventing the invasion of pathogens (Basavaprabhu et al., 2020).

Srinivasan et al. reported that microbiomes abundant in *L. crispatus* were consistently strongly associated with low vaginal pH, but this was not the case for women with *L. iners* overgrowth (Srinivasan et al., 2012). In fact, *L. iners* was very weak in a vaginal environment with low pH, failing to maintain intravaginal acidity. A high vaginal pH is a characteristic of BV, a condition in which *Gardnerella vaginalis* and *L. iners* are generally found in the vaginal econiche but other *Lactobacillus* species are rarely found (Muzny et al., 2018; Pleckaityte, 2019). In addition, *L. iners* does not have the molecular and cellular machinery to produce H_2_O_2_ through pyruvate oxidation. The production of H_2_O_2_ is considered to be one of the mechanisms by which *Lactobacillus* species can prevent anaerobic bacteria from colonizing the vagina (Felten et al., 1999; Ojala et al., 2014). As such, when pathogenic bacteria challenge the vaginal environment, *L. iners* cannot resist the overgrowth of pathogenic bacteria and the increase of pH, whereas it may persist in dysbiosis (Chee et al., 2020).

### Adhesive Capability of *L. iners*

The adherence of vaginal *Lactobacillus* species to host cells is believed to play a role in the exclusion of pathogenic microorganisms through a mechanism that involves the blocking of their binding sites on vaginal epithelial cells (Pino et al., 2019; Mane et al., 2020). Although *L. iners* lacks most of the main adhesion molecules of *Lactobacillus* species (Morris et al., 2012), it still shows a strong adhesive ability to vaginal epithelial cells (McMillan et al., 2013). Fibronectin is an insoluble glycoprotein in the extracellular matrix of the vaginal epithelium (Park et al., 2012). The *L. iners* genome encodes a fibronectin-binding protein that contains a motif (fibronectin-binding protein A) common to pathogenic strains of *Staphylococcus aureus*, thereby mediating the adhesion and the invasion of *S. aureus* to host cells (Macklaim et al., 2011; Macklaim et al., 2013). McMillan et al. demonstrated that *L. iners* bound significantly stronger to human fibronectin than other *Lactobacillus* species at a more neutral pH, which may contribute to the persistence of *L. iners* in the vagina despite the presence of pathogens or treatment with antibiotics (McMillan et al., 2013). An *in vitro* study reported that *L. iners* may increase the adhesion of BV-causing *G. vaginalis* (Castro et al., 2013). It was also demonstrated that *L. iners* produces inerolysin, a pore-forming protein typically found in pathogenic bacteria, which can enhance the adhesive ability (Rampersaud et al., 2011; Ragaliauskas et al., 2019). These findings indicate that the unique adhesive function of *L. iners* reduces the protection of the healthy vaginal microbiome from pathogenic bacteria.

### Requirement of Nutrients From Exogenous Sources

*L. iners* has an unusually small genome with reduced metabolic capabilities, but it contains a broader array of genes that was probably acquired from foreign sources. The nutrient requirements of this species are more complex than those of other vaginal *Lactobacillus* species, thereby allowing *L. iners* to adapt to the diverse niche in the vagina (Macklaim et al., 2011). The fluctuation of hormones and other factors may affect the vaginal environment, resulting in changes in mucus and glycogen production, pH, and microbial species, which may provide essential nutrients for *L. iners* (Kwak et al., 2020). Genome analysis has indicated a higher dependence of *L. iners* on exogenous sources of amino acids (France et al., 2016). Furthermore, *L. iners* has the molecular and cellular machinery to ferment glucose, maltose, trehalose, and mannose, among which glucose and maltose are common glycogenolysis products (France et al., 2016). Macklaim et al. reported that *L. iners* genes for the uptake of mannose and maltose, genes for glycogen decomposition, as well as genes for mucin and glycerol transport, were strongly up-regulated in BV (Macklaim et al., 2013). Although no iron uptake system has been identified in *L. iners*, ferrochelatase, which is capable of catalyzing ferrous ion and binding protoporphyrin IX to form heme, was detected in *L. iners* (Macklaim et al., 2011).

The ability of *L. iners* to produce inerolysin may be one of the most important factors influencing its ability to acquire nutrients from the vaginal environment. *L. iners* is the only *Lactobacillus* species known to code for inerolysin, which is related to intermedilysin (69.2% similarity) and vaginolysin (68.4% similarity) produced by *Streptococcus intermedius* and *G. vaginalis*, respectively (Rampersaud et al., 2011). Over 10% of genes coding for inerolysin in *L. iners* are more highly expressed in dysbiosis than in balanced microbial environments (Macklaim et al., 2013). This cytolysin can liberate resources directly from host tissues or cells, which necessitates that *L. iners* acquire its nutrients from the host in a symbiotic way (Macklaim et al., 2011). In other words, this characteristic may give *L. iners* a competitive advantage in the vaginal environment when nutrients are scarce, especially under potentially adverse conditions, such as BV, when other *Lactobacillus* species cannot colonize the vagina (Zozaya-Hinchliffe et al., 2010; Li and Ma, 2020).

## *L. iners* and Women Diseases

### *L. iners* and Vaginal Dysbiosis

Compared with intestinal microflora, a typical feature of the vaginal microbial environment in healthy individuals is its extremely low bacterial diversity (Ravel et al., 2011; Collins et al., 2018). There are five major community-state types (CSTs) in healthy premenopausal women, namely, *L. crispatus*-dominated CST I, *L. gasseri*-dominated CST II, *L. iners*-dominated CST III, and *L. jensenii*-dominated CST V, whereas CST IV is characterized by the absence of *Lactobacillus* species (Wells et al., 2020). Vaginal dysbiosis, which is defined by a high bacterial diversity and a mixture of anaerobic bacteria, is frequently associated with a variety of gynaecological diseases (Eastment and McClelland, 2018; Chee et al., 2020; Chen et al., 2021).

*L. iners* can be predominant in the vagina of healthy women, or in those with vaginal dysbiosis, such as BV, or even in those receiving antimicrobial therapy (Ferris et al., 2007; Goodfellow et al., 2021). Many studies have reported that the presence of *L. crispatus* in the vagina is associated with good health, whereas communities dominated by *L. iners* fail to provide sufficient protection against vaginal dysbiosis (Petricevic et al., 2014; France et al., 2016; Tortelli et al., 2020). The existence of *L. iners* is related to higher levels of proinflammatory factors, such as interleukin-1α, interleukin-18, macrophage migration inhibitory factor, and tumor necrosis factor-α, which are responsible for the activation of an inflammatory response in the vagina (De Seta et al., 2019). *L. iners* is even believed to play a role in the onset of vaginal dysbiosis (Petrova et al., 2017), although the precise role of *L. iners* remains debated. However, it seems that the abundance of *L. iners* remains relatively constant, and *L. iners* is not easily displaced by pathogens or infectious conditions. In cases of BV, *L. iners*, rather than *L. crispatus*, usually coexists with other potentially harmful bacteria that colonize the vagina (Ferris et al., 2007; Zozaya-Hinchliffe et al., 2010). The ability of *L. iners* to adapt to dysbiosis, despite its small genome, may be related to its changes in genes involved in metabolism and cytolysis, as well as antibacteriophage defense genes, to changing conditions in the vagina (Borgdorff et al., 2016; Leizer et al., 2018). The remarkable ability of *L. iners* to survive under various conditions indicates that this species may be an important member of the host’s defense and may be a persistent symbiotic *Lactobacillus* species that can maintain and restore the vaginal microbiome (Macklaim et al., 2011).

### *L. iners* and BV

BV is the most common type of vaginitis in women of childbearing age. It is characterized by a significant reduction or disappearance of *Lactobacillus* species, accompanied by the emergence of more diverse microbiota dominated by anaerobic and facultative bacteria such as *Gardnerella* species, *Prevotella* species, and *A. vaginae* (Fredricks et al., 2005; Lee et al., 2020; Witkin et al., 2021). However, *L. iners* is usually the only vaginal *Lactobacillus* species coexisting with BV-associated bacteria that can be detected during BV (Macklaim et al., 2011; Santiago et al., 2012). It can persist under the drastically changing vaginal environment of BV due to its ability to respond and regulate its genomic functions (Macklaim et al., 2013). The increased gene expression of *L. iners* may lead to the production of succinate and other short-chain fatty acids and the increase in the pH value in the BV environment (Macklaim et al., 2013). To adapt to the BV environment, *L. iners* can increase the expression of inerolysin and mucin, and promote the production of glycerol and the expression of related metabolic enzymes, which ensures its acquirement of nutrients from foreign sources (Macklaim et al., 2013). In addition, bacteriophages were one of the reasons for the sudden decrease of *Lactobacillus* species during BV, whereas *L. iners* can upregulate defense systems such as the type I RM system and CRISPR, as well as its specific hsdR gene, to resist bacteriophage invasion during BV (Kwak et al., 2020). Nevertheless, a recent study discovered three active peptides of bacteriocin produced by a human intestinal strain named *Lactobacillus paragasseri*. These bacteriocins have strong selective inhibitory activity against *L. iners*, whereas *L. crispatus*, *L. jensenii*, and *L. gasseri* were only slightly inhibited, indicating that these *Lactobacillus*-derived effective inhibitors of *L. iners* can be combined with metronidazole to improve the current BV treatments (Nilsen et al., 2020).

As the coexistence of *L. iners* in BV is different from that of other *Lactobacillus* species, the prevalence of *L. iners* can be used as a microbial indicator to predict the onset of BV or the intermediate BV status (Basavaprabhu et al., 2020). Furthermore, *L. iners* is metronidazole-resistant, and it was found to be the predominant *Lactobacillus* species, even after treatment of BV with metronidazole (Ferris et al., 2007; Srinivasan et al., 2010; Mayer et al., 2015; Lehtoranta et al., 2020). Compared with other more protective *Lactobacillus* species, which hardly exist during BV, *L. iners* showed a stronger competitive advantage and coexisted in the disrupted microbiome (Nilsen et al., 2020). It has been proposed that *L. iners* facilitates the transition between BV and non-BV states (Shipitsyna et al., 2013; Petrova et al., 2015). Interestingly, it has been reported that even after BV treatment, the vaginal microbiome does not change from the *L. iners*-dominant state to the *L. crispatus*-dominated state (Lambert et al., 2013). Therefore, the persistence of *L. iners* may lead to long-term vaginal dysbiosis, especially after repeated treatment cycles of BV (Nilsen et al., 2020). Further studies are needed to clarify whether this species is only a biomarker of the vaginal microbiota transition or a contributing factor of BV.

### *L. iners* and Biofilm Formation

Biofilms are bacterial structures tightly attached to a surface, and they are known to be more resistant to the host immune response and antibiotic therapy than planktonic cells (Hall-Stoodley et al., 2012). It has been shown that biofilm formation on vaginal epithelial cells is strongly associated with vaginal infections (Costerton et al., 1999; Srinivasan and Fredricks, 2008). There is sufficient evidence that BV associates with the presence of a dense polymicrobial biofilm, in which *G. vaginalis* is the dominant bacterial strain on the vaginal epithelium (Machado et al., 2015; Rosca et al., 2020). It has been hypothesized that *Gardnerella* spp. initiate biofilm formation, which supports the attachment of other BV-associated bacteria (BVAB) to the vaginal epithelium, further enhancing the biofilm thickness (Muzny et al., 2019). Moreover, *Gardnerella* biofilms serve as barriers to antibiotics and function to protect other BVAB by preventing the penetration of antibiotics (Gustin et al., 2021). It is generally believed that the high rate of BV recurrence is due to the formation of biofilms that protect the bacteria from antibiotic treatment, and even serve as a reservoir for pathogen regrowth (Bradshaw et al., 2006; Gottschick et al., 2017).

Vaginal indigenous *Lactobacilli* are believed to prevent the colonization of pathogenic bacteria through steric hindrance or receptor masking in the mucosa (Zarate and Nader-Macias, 2006). Previous studies have used a *Lactobacillus* probiotic approach in an attempt to clear the polymicrobial biofilms, essentially impeding bacterial virulence and suppressing infection in the human vagina (Saunders et al., 2007; Hardy et al., 2017; Chee et al., 2020). *Lactobacillus plantarum* was reported to significantly reduce the adhesion of *Escherichia coli*, *Salmonella typhimurium*, *Staphylococcus aureus*, and *Pseudomonas aeruginosa* in the HT-29 cell line, which made it a potential anti-biofilm agent for BV treatment (Liu et al., 2017). Saunders et al. reported that *G. vaginalis* biofilms grown *in vitro* were displaced with *Lactobacillus reuteri* RC-14, and to a limited extent with *L. iners* (Saunders et al., 2007). Castro et al. demonstrated that *L. crispatus* drastically reduced the adhesion of *G. vaginalis* strains, both from a healthy woman and a woman with BV, to cervical epithelial cells. Interestingly, *L. iners* significantly reduced the adhesion of *G. vaginalis* strains from a healthy woman, but markedly enhanced pathogenic *G. vaginalis* adhesion (Castro et al., 2013), suggesting that *L. iners* can cohabitate with BV-associated *G. vaginalis* and may contribute to *G. vaginalis*-dominated biofilm formation (Gottschick et al., 2017). In addition, it is well known that *Candida* species, mainly *C. albicans*, can form thick and tough biofilms, which greatly increases the tolerance to antifungal drugs during the treatment of recurrent vulvovaginal candidiasis (Taff et al., 2013). Mckloud et al. reported the ability of various *Lactobacillus* species to inhibit *C. albicans* biofilm formation and biofilm-related gene expression when cocultured (McKloud et al., 2021). *Lactobacillus rhamnosus* could down-regulate *C. albicans* biofilm-related gene expression. Conversely, coculture with *L. iners* resulted in an up-regulation of biofilm-related gene expression (ALS3 and ECE1), suggesting that the presence of *L. iners* may be indicative of a shift to vaginal dysbiosis; therefore, it should not be used as a probiotic intervention for *C. albicans* infection (Ponomarova et al., 2017). A further understanding of the interactions between vaginal commensal *Lactobacilli* and the structure and function of biofilms is of extreme importance to identify novel treatment approaches for biofilm-associated infections (Falconi-McCahill, 2019).

### *L. iners* and STIs

Previous studies have reported that *L. crispatus*-dominated vaginal microbiomes associate with a lower prevalence of STIs, whereas BV associates with an elevated risk of STIs such as infection with *Chlamydia trachomatis*, human immunodeficiency virus (HIV), *Neisseria gonorrhoeae*, cytomegalovirus, and herpes simplex virus-2 (Bayigga et al., 2019; Gondwe et al., 2020; Redelinghuys et al., 2020). Van Houdt et al. reported that the vaginal microbiome dominated by *L. iners* at baseline significantly increased the risk of acquiring *C. trachomatis* infection one year later (van Houdt et al., 2018). A lack of D-lactic acid in the *L. iners*-dominated vaginal microbiome may increase the ability of HIV to transverse the cervicovaginal mucus by modulating cervical integrity (Witkin, 2015; Reimers et al., 2016; Hoang et al., 2020). Interestingly, Mehta et al. and Reimers et al. demonstrated that the vaginal microbiome did not differ between HIV-positive and HIV-negative black women in the United States (Mehta et al., 2015; Reimers et al., 2016). However, Spear et al. conversely observed that the percentage of *L. iners* was significantly higher in HIV-negative African Americans than in HIV-positive African Americans (Spear et al., 2011). The reason for these inconsistent results may be differences in the genetic background or complicated social and behavioral factors in black women, as black women without BV were more likely to have vaginal microbiomes dominated by *L. iners* (Fettweis et al., 2014; Wells et al., 2020). The precise role of *L. iners* in HIV infection should be further examined. Many studies reported a higher diversity of vaginal microbes and a lower abundance of *Lactobacillus* species among HPV-positive women (Lee et al., 2013; Oh et al., 2015; Reimers et al., 2016). Norenhag et al. showed that the vaginal microbiome dominated by *L. iners* was associated with high-risk HPV infection compared with *L. crispatus* (Norenhag et al., 2020). It can be speculated that vaginal dysbiosis may affect the host’s innate immunity against HPV infection, resulting in dysplasia/cervical cancer (Kyrgiou et al., 2017). These findings indicate that *L. iners* may exhibit rapid changes in the composition of the vaginal microbiome similar to BV and could be a valuable biomarker of the dynamic vaginal environment under STIs (Ravel et al., 2013; van Houdt et al., 2018).

### *L. iners* and Preterm Birth (PTB)

It is especially important to maintain the natural and healthy balance of *Lactobacillus* species in the vaginal microbiome during pregnancy (Zheng et al., 2019; Juliana et al., 2021). Earlier studies confirmed that high estradiol levels and the consequent high glycogen levels in the vagina during pregnancy result in stronger vaginal acidification, thereby promoting the prevalence of *Lactobacillu*s species as gestation progresses (Aagaard et al., 2012; Basavaprabhu et al., 2020). However, many studies have indicated that the *L. iners*-dominated vaginal microbiome was more likely to shift towards dysbiosis during pregnancy (Mls et al., 2019; Kumar et al., 2021; Sarmento et al., 2021). In our previous study, we found that the abundance of *L. iners* decreased significantly in the second and third trimester, whereas that of *L. crispatus* increased in the second trimester compared with the first trimester in healthy pregnant women (Zheng et al., 2019). In addition, we observed that the increase in the abundance of *L. iners*, but not that of *L. crispatus*, was related to the increase in vaginal cleanliness and positive leukocyte esterase activity, which is consistent with the results of a previous study (Vaneechoutte, 2017).

Increasing evidence indicates that BV is one of the major etiological causes for adverse pregnancy outcomes, especially PTB (Guerra et al., 2006; Basavaprabhu et al., 2020; Redelinghuys et al., 2020). The *L. iners*-dominated vaginal microbiome, a so-called ‘intermediate microflora’ and a typical feature of BV, is speculated to be a risk factor for PTB (Petricevic et al., 2014; Kindinger et al., 2017). Petricevic et al. suggested that the prevalence of *L. iners* detected in vaginal smears of healthy women in early pregnancy can associate with PTB (Petricevic et al., 2014). This was also demonstrated by Kindinger et al., who reported that the vaginal microbiome dominated by *L. iners* at 16 weeks of gestation is a risk factor for both a short cervix and early PTB (<34 weeks), whereas *L. crispatus* dominance is protective against PTB in a more ethnically diverse cohort (Kindinger et al., 2017). Recent studies from different countries also showed a significant association between *L. iners* and an increased prevalence of PTB (Elovitz et al., 2019; Aslam et al., 2020; Goodfellow et al., 2021; Kumar et al., 2021; Payne et al., 2021; Sarmento et al., 2021). However, several studies reported no significant association between *L. iners* and PTB (Callahan et al., 2017; Blostein et al., 2020; Witkin et al., 2021). *L. iners* was also demonstrated to be the most abundant *Lactobacillus* species among pregnant black women (Wells et al., 2020). However, most studies did not identify a significant relationship between the *Lactobacillus*-dominant vaginal microbiome and PTB in pregnant black women (Hyman et al., 2014; Nelson et al., 2016; Subramaniam et al., 2016; Stout et al., 2017). Conversely, three studies reported that *L. iners* was associated with a decreased risk of PTB (Fettweis et al., 2019; Tabatabaei et al., 2019; Park et al., 2021). Therefore, the association between *L. iners* and PTB risk is controversial (**Table 1**). Presently, it is believed that the vaginal microbiome in black women does not play an important role in the pathogenesis of PTB, as it does in Caucasians and Asians (Kindinger et al., 2017). Furthermore, the limited sample size, the time of sample collection, differences in the definition of PTB, ethnical and geographical variations, and differences in the methodology of strain identification, as well as complicated clinical conditions such as genetic abnormalities or a history of PTB, were confounding factors that impacted the results (Ravel et al., 2011; Jespers et al., 2012; Mehta et al., 2020; Wells et al., 2020).

**Table 1:** Main results per study on *L. iners* and PTB.

Author (year)	Country	Time of sample collection	Sample size	Tools implied for identification	Main findings	References	
Petricevic et al.	Austria	At 11-14 weeks of gestation	111 women (white European, Middle Eastern, Asian)	PCR-DGGE and sequencing	*L. iners* was the predominant vaginal *Lactobacillus* spp. in women who delivered preterm newborns. *L. iners* was predominantly present in 11/13 (85%) of women who delivered preterm newborns and in only 16/98 (16%) of women who delivered at term (p < 0.001).	(Petricevic et al., 2014)	
Kindinger et al.	United Kingdom	At 16 weeks of gestation	161 women (30 Black, 104 Caucasian, 27 Asian)	16S rRNA gene sequencing at V1-V3 region	*L. iners* dominance of the vaginal microbial community at 16 weeks of gestation was significantly associated with both a short cervix <25 mm and early PTB (<34 weeks). By contrast, *L. crispatus* dominance was highly predictive of TB.	(Kindinger et al., 2017)	
Callahan et al.	United States	Weekly	Low-risk cohort: 39 women; High-risk cohort: 96 women	16S rRNA gene sequencing at V4 region	*L. crispatus* was associated with the low risk of PTB in low- and high-risk cohorts, whereas no significant association was detected for *L. iners*. A subspecies clade of *Gardnerella vaginalis* explained the genus association with PTB.	(Callahan et al., 2017)	
Tabatabaei et al.	Canada	At 8-13 weeks of gestation	94 spontaneous PTB cases, 356 term controls	16S rRNA gene sequencing at V4 region	*Lactobacillus gasseri*/*L. johnsonii*, *L. crispatus* (99%)/*L. acidophilus* (99%), *L. iners* (99%)/*Ralstonia solanacearum* (99%) and *Bifidobacterium longum*/*Bifidobacterium breve* were associated with a decreased risk of early but not late spontaneous PTB.	(Tabatabaei et al., 2019)	
Elovitz et al.	United States	At 16–20 weeks, 20–24 weeks and 24–28 weeks of gestation	539 women (402 African American, 115 white, 22 other)	16S rRNA gene sequencing at V3-V4 region	In non-African American women, *L. iners* and *A. vaginae* were significantly associated with increased rates of spontaneous PTB.	(Elovitz et al., 2019)	
Fettweis et al.	United States	At prenatal visit, at triage	45 spontaneous PTB cases and 90 term controls (African American predominantly)	16S rRNA gene sequencing at V1-V3 region	*L. crispatus* increased in abundance during pregnancy in women who delivered preterm newborns. Women who delivered at term exhibited significant decreases in the abundance of *A. vaginae* and *G. vaginalis*, and an increase in the abundance of *L. iners*.	(Fettweis et al., 2019)	
Aslam et al.	Pakistan	Not available	8 term vaginal swabs, 8 preterm vaginal swabs, and 8 preterm placenta tissues	16S rRNA gene sequencing at V1-V2 region	Metagenomics data of vaginal swabs and placental tissues from severe PTB indicated that *L. iners* was the main difference between term and preterm deliveries. Overall, the lack of *Lactobacillus* species or the presence of rogue *Lactobacillus* species, such as *L. iners* and *L. vaginilis*, was associated with PTB.		(Aslam et al., 2020)
Blostein et al.	Peru	Before 16 weeks of gestation (9 weeks on average)	25 PTB cases and 100 term controls	16S rRNA gene sequencing at V4 region	Overall, no CST (diverse, *Lactobacillus*-dominated, or *L. iners*-dominated) was associated with PTB in crude or adjusted logistic models, whereas women with *Lactobacillus*-dominated CSTs were less likely to have PTB than those with diverse CST among women sampled before 12 weeks of gestation.		(Blostein et al., 2020)
Sarmento et al.	Brazil	In the second trimester	146 women	16S rRNA gene sequencing at V1-V3 region	*L. iners* was the dominant vaginal bacterium in 61.5% of women with spontaneous PTB but only in 31.2% of those who delivered at term (p = 0.035).		(Sarmento et al., 2021)
Kumar et al.	India	In each trimester of pregnancy	18 PTB cases and 20 term controls	16S rRNA gene sequencing at V3-V4 region	A significantly higher abundance of *L. iners* (all trimesters), *Megasphaera* sp (first trimester), *Gardnerella vaginalis* (second trimester), and *Sneathia sanguinegens* (second trimester) was identified in preterm samples, whereas a higher abundance of *L. gasseri* (third trimester) was observed in term samples.		(Kumar et al., 2021)
Witkin et al.	Brazil	In the second trimester	613 women	16S rRNA gene sequencing at V1-V3 region	Spontaneous PTB occurred in 9.6%, 9.3%, and 6.9% of women when *G. vaginalis*, *L. iners*, or *L. crispatus* was the dominant species, respectively, but the differences were not statistically significant.		(Witkin et al., 2021)
Goodfellow et al.	United Kingdom	At 15-22 weeks of gestation	109 high-risk women and 145 low-risk women	16S rRNA gene sequencing at V3-V4 region	*L. iners* achieved higher bacterial loads compared to the other *Lactobacillus* species and associated with early spontaneous PTB/PPROM recurrence.		(Goodfellow et al., 2021)
Park et al.	Korea	At 15-34 weeks of gestation	38 PTB cases and 56 term controls	Multiplex quantitative real-time PCR	Although most values for single bacteria were not statistically significant, the mean value of the total Bacillus class showed a significant difference between PTB and TB groups, in which the mean value of *L. iners* showed a significant increase in the TB group.		(Park et al., 2021)
Payne et al.	Australia	At 12-23 weeks of gestation	936 women (white race predominantly)	Quantitative PCR	Women who delivered at term had a higher level of *L. crispatus*, *L. gasseri*, or *L. jensenii* DNA in their vaginal swabs. In the remaining women, a specific microbial DNA signature was identified, which was strongly predictive of spontaneous PTB risk, consisting of *G. vaginalis*, *L. iners*, and *Ureaplasma parvum* DNA.		(Payne et al., 2021)

PTB, preterm birth; TB, term birth; PPROM, preterm prelabor rupture of membranes; DGGE, denaturing gradient gel electrophoresis.

According to the features of *L. iners* and its lack of protection against pathogens when it is the only *Lactobacillus* species in the vagina, some researchers have suggested that *L. iners* cannot be responsible for infections during pregnancy (Petricevic et al., 2014; Peelen et al., 2019). Indeed, because the vaginal microbiome dominated by *L. iners* is relatively unstable, there is a tendency for transition to BV-associated CST-IV during pregnancy (Verstraelen et al., 2009). In addition, the *L. iners*-dominated vaginal microbiome may increase the risk of PTB by modulating local tissue inflammation and cervical integrity, thereby disrupting chemical and mechanical mucosal protective barriers against ascending infections (Kindinger et al., 2017). Further studies are needed to clarify the potential mechanisms between the *L. iners*-dominated vaginal microbiome and PTB.

### *L. iners* and Infertility

Previous studies have reported that up to 40% of patients who failed assisted reproduction by *in vitro* fertilization (IVF) had an abnormal reproductive tract microbiome (Fanchin et al., 1998; Moore et al., 2000; Koedooder et al., 2019). Vaginal dysbiosis, including an elevated pH value, increased flora diversity, BV, vulvovaginal candidiasis, and trichomonal vaginitis, are recognized as risk factors for infertility (Campisciano et al., 2017; Moumne et al., 2021). Campisciano et al. reported that the abundance of *L. iners* was associated with an increased infertility rate (Campisciano et al., 2020). Chen et al. also recently reported that the *L. iners*-dominated vaginal microbiome was associated with tubal infertility and *C. trachomatis* infection (Chen et al., 2021). As a transitional species, *L. iners* may facilitate the transition between an abnormal and a normal vaginal microbiome under treatment or artificially high estrogen levels that are needed for IVF (Kindinger et al., 2017; Kosti et al., 2020). It is believed that the *L. iners*-dominated vaginal microbiome is an unfavorable factor for pregnancy.mk

## *L. iners* and the Menstruation Cycle

The Human Microbiome Project shows that in the microbial community for all body parts, including the vagina, within-subject variations over time are consistently lower than between-subject variations (Human Microbiome Project, 2012). The menstrual cycle is one of the most important factors disturbing the diversity of the vaginal microbiome (Chaban et al., 2014; Chen et al., 2021). *L. crispatus* usually dominates the vagina of reproductive-age women, whereas *L. iners* overgrows and replaces *L. crispatus* during the menstruation cycle (Gajer et al., 2012; Santiago et al., 2012). A recent study reported that *L. iners* was the most recurrent microbe in the follicular phase; *L. iners* and CST IV (microbial diversity) were predominant in the periovulatory phase; and in the luteal phase, the most frequent type was CST IV (Alonzo Martinez et al., 2021). Indeed, the abundance of *L. iners* remarkably increases during menses, frequently in conjunction with an increase of *G. vaginalis* and/or *Atopobium vaginae*; however, they subsequently decrease after menses without intervention (Jespers et al., 2012; Petrova et al., 2015). As dynamic changes in the vaginal econiche were characterized at different time points in the menstruation cycle within the same individual, the moment of sampling relative to the menstrual cycle is very important for vaginal community analysis.

## Anti-Microbial and Immune-Inducing Activity of *L. iners*

*L. iners* is the most common and persistent vaginal symbiotic *Lactobacillus* species with good adaptability to the complex and dynamically changing vaginal environment (Borgdorff et al., 2016; Kwak et al., 2020). Under fluctuating environmental conditions, other *Lactobacillus* species may not survive, while *L. iners* persists with relatively constant abundance due to its ability to respond and regulate its genomic functions, such as specific carbohydrate uptake, fibronectin-binding protein activity, bacteriophage defense, and inerolysin synthesis (Rampersaud et al., 2011; Macklaim et al., 2013; McMillan et al., 2013; Kwak et al., 2020). This remarkable ability to survive under a range of conditions contributes to *L. iners*’ being a dominant species when the microbiome is in a transitional stage (Jakobsson and Forsum, 2007), suggesting that *L. iners* may be an important member of the host defense mechanism as a persistent mutualistic lactobacilli, and even promote the restoration of a healthy vaginal microbiome (Ravel et al., 2011; France et al., 2016).

In fact, several studies have confirmed many probiotic characteristics of *L. iners*. It has many ecological functions, such as lactic acid production, that are similar to those of other *Lactobacillus* species (Linhares et al., 2011; O’Hanlon et al., 2011). Although the genome of *L. iners* lacks most of the adhesion factors of other *Lactobacillus* species, it can still adhere strongly to vaginal epithelial cells (Morris et al., 2012; McMillan et al., 2013).Thus, *L. iners* shows the similar phenotypic traits of colonization and host interaction, as well as excluding pathogens, as other vaginal *Lactobacillus* species (Osset et al., 2001). Shipitsyna et al. reported the loss of *L. iners* during BV and suggested that it was not the key pathogen causing the disease (Shipitsyna et al., 2013). *L. iners* can destroy or replace *G. vaginalis* to form biofilms *in vitro* (Hummelen et al., 2010; Zhou et al., 2010). Macklaim et al. demonstrated that some specific functions of *L. iners*, such as the expression of cytokines, absorption of exogenous sources, and bacteriophage defense, facilitated the harsh conditions in the vagina, including BV (Macklaim et al., 2013). *L. iners* prevents harmful bacteria from obtaining important nutrients, such as iron, and inhibits their sustained growth by triggering the innate immune system in vaginal epithelial cells (Vaneechoutte, 2017). In addition, the anti-inflammatory effects of *L. iners* were observed through specific molecular interactions between vaginal epithelial cells (Rose et al., 2012).

The *L. iners*-dominated vaginal microbiome was associated with the induction of a stress response in the vaginal epithelium (Vaneechoutte, 2017; Linhares et al., 2019). Doerflinger et al. discovered that *L. iners*, but not *L. crispatus*, significantly upregulated the pattern-recognition receptor signaling pathway in human primary vaginal epithelial cells and increased the mRNA expression of tumor necrosis factor, indicating that the vaginal microbiome regulates the host immune response species-specifically (Doerflinger et al., 2014). It has been suggested that, in response to stress, *L. iners* can activate the toll-like receptor signaling pathway in vaginal epithelial cells, increase heat shock protein 70 expression, and inhibit autophagy, which would destroy the homeostasis between vaginal epithelial cells and reduce the ability of these cells to recognize and respond to potential pathogens (Doerflinger et al., 2014; Feng et al., 2015). Conversely, many compounds involved in the antimicrobial defense of vaginal epithelial cells, such as neutrophil gelatinase-associated lipocalin, calprotectin, and hyaluronan, were also preferentially induced by *L. iners* (Leizer et al., 2018). These findings support the fact that *L. iners* can fight non-physiological threats, and maintain and promote the recovery to a healthier state, as well as exhibit proinflammatory qualities and act less like a commensal microbe under different conditions (Levine et al., 2011).

## Conclusions

In conclusion, *L. iners* is a unique and intriguing *Lactobacillus* species with extraordinary characteristics. Its small genome and concurrent nutrient dependency are conducive to its high adaptation to both the low and the high pH vaginal environment, as well as both BV-positive and BV-negative conditions. Therefore, *L. iners* is often classified as a transitional species that colonizes the vagina after an ecological disturbance. However, whether *L. iners* is beneficial or pathogenic for the host’s microbiome remains controversial. Most researchers are inclined to believe that *L. iners* offers limited protection against vaginal colonization by pathogens and may contribute to the onset and maintenance of vaginal dysbiosis. *L. iners* may also be a risk factor for sexually transmitted infections and adverse pregnancy outcomes. A greater understanding of the roles of *L. iners* in health and diseases in individuals of different races and ethnicities is warranted. In addition, further studies are required to clarify the role of *L. iners* in vaginal mucosal immune regulation, and to further clarify whether it can be used as a novel biomarker to detect the existence or prognosis of vaginal inflammation and guide subsequent clinical treatment.

## Author Contributions

NZ, RG, JW, WZ, and ZL discussed the contents, wrote, reviewed and edited the manuscript. All authors contributed to the article and approved the submitted version.

## Funding

This present work was funded by the grants of the National Natural Science Foundation of China (81771724, 31700800, 81790631), the Taishan Scholar Foundation of Shandong Province (tsqn202103119), the Nutrition and Care of Maternal & Child Research Fund Project of Guangzhou Biostime Institute of Nutrition & Care (2019BINCMCF045), the National S&T Major Project of China (2018YFC2000500), and the Foundation of China’s State Key Laboratory for Diagnosis and Treatment of Infectious Diseases.

## Conflict of Interest

The authors declare that the research was conducted in the absence of any commercial or financial relationships that could be construed as a potential conflict of interest.

## Publisher’s Note

All claims expressed in this article are solely those of the authors and do not necessarily represent those of their affiliated organizations, or those of the publisher, the editors and the reviewers. Any product that may be evaluated in this article, or claim that may be made by its manufacturer, is not guaranteed or endorsed by the publisher.
